# Gestational weight gain in pregnant women with obesity is associated with cord blood DNA methylation, which partially mediates offspring anthropometrics

**DOI:** 10.1002/ctm2.1215

**Published:** 2023-03-16

**Authors:** Josefine Jönsson, Kristina M. Renault, Alexander Perfilyev, Allan Vaag, Emma Malchau Carlsen, Kirsten Nørgaard, Paul W. Franks, Charlotte Ling

**Affiliations:** ^1^ Department of Clinical Sciences Epigenetics and Diabetes Unit Lund University Diabetes Centre Scania University Hospital Lund University Malmö Sweden; ^2^ Department of Obstetrics and Gynecology Hvidovre Hospital University of Copenhagen Copenhagen Denmark; ^3^ Department of Obstetrics Juliane Marie Centret Rigshospitalet University of Copenhagen Copenhagen Denmark; ^4^ Steno Diabetes Center Copenhagen Gentofte Denmark; ^5^ Department of Nutrition Exercise and Sports Faculty of Science University of Copenhagen Frederiksberg Denmark; ^6^ Department of Pediatrics Copenhagen University Hospital Hvidovre Hvidovre Denmark; ^7^ Department of Clinical Sciences Genetic and Molecular Epidemiology Unit Lund University Diabetes Centre Lund University Malmö Sweden

**Keywords:** birthweight, epigenetics, fetal programming, lean mass

Dear Editor,

Obesity and excessive gestational weight gain (GWG) influence the offspring's future health, and DNA methylation, an epigenetic mark occurring on CpG sites, constitutes a potential mechanism underlying this relationship.^1^, ^2^ GWG has been associated with birthweight, fat mass in newborns, and childhood obesity.^1^ Additionally, other suggested offspring long‐term health effects linked to GWG are cancers and neurodevelopmental outcomes such as attention‐deficit/hyperactivity disorder.^1^ To our knowledge, associations between GWG and DNA methylation in cord blood have only been investigated in normal‐weight populations using either epigenome‐wide association studies (EWAS)^3^, ^4^ or a panel of ∼1500 sites^5^. In the EWAS of normal‐weight women, no associations between GWG and DNA methylation in cord blood were found.^3^, ^4^ We, therefore, examined if GWG in women with obesity, with a wide range of GWG (‐5.0–34.1kg), associated with cord blood DNA methylation in the offspring of 232 newborns participating in the Treatment of Obese Pregnant Women study using EWAS (Figure S1 and Table 1, see Methods in Supporting Information). Approximately half of the women exceeded, and a quarter either stayed within or gained less weight than the Institute of Medicine recommendations regarding GWG. To test if cord blood DNA methylation is associated with GWG independent of treatment allocation, a linear regression model adjusted for maternal and offspring confounders and cell‐type composition using a reference‐free method was used (Model 1, Figure 1A). We additionally examined three models: Model 2, an unadjusted model without cell‐type composition adjustment; Model 3, adjusted for maternal and offspring confounders, without cell‐type composition adjustment; and Model 4, adjusted for maternal and offspring confounders and cell‐type composition using a reference‐based method (Figure 1A, see details in Supporting Information, Methods). In model 1, we found GWG to associate with differential DNA methylation at 441 sites, annotated to 352 genes, for example, *ABCC8*, *FOXA2*, *GATA3*, *GRB10*, *NEUROD2, SMAD2*, and *TUB* in cord blood based on a false discovery rate [FDR] <5% (Model 1; Figure 1A,B and Table S1). Six sites remained statistically significant after the Bonferroni correction (Figure 1B–H). DNA methylation of 13 of these 441 sites was also associated with gestational age (GA) and one with the lifestyle intervention, while none was associated with age, body mass index (BMI), or sex (FDR<5%, Table S1). Additionally, one of our found GWG‐associated methylation sites, cg14663510 (*HMX3*), has previously been linked to a low pre‐pregnancy BMI (Table S1)^3^. GWG was associated with methylation at 410, and 413 of the 441 sites in Model 2 and 3, respectively, and all 441 methylation sites were nominally associated with GWG in Model 4 (Figure 1A and Table S1). We further validated the results from Model 1 by randomly splitting the cohort into a discovery and validation cohort (60:40). In the discovery cohort (*n* = 125), 438 of our 441 methylation sites (99%) associated with GWG based on P = 8.77 × 10^−9^–4.00 × 10^−2^, while in the validation cohort (*n* = 83), 328 of our 441 methylation sites were identified with P = 1.98 × 10^−10^–4.9 × 10^−2^ (Table S1). Furthermore, 74% of the sites found in the discovery cohort could be confirmed in the validation cohort. Our results differ from previous studies performed on normal‐weight women.^3^, ^4^, ^5^ These discrepancies may be due to differences in maternal BMI (normal‐weight vs. obese), large variations in GWG in our study, different methods for cell‐type composition adjustment, and different time points for measuring GWG.

**TABLE 1:** Parental and offspring baseline characteristics for subjects with available cord blood of the Treatment of Obese Pregnant Women (TOP) study

Maternal characteristics	*n* = 208
Maternal age at enrolment (years)^†^	31.07 (4.45)
Pre‐pregnancy BMI (kg/m^2^)^†^	34.25 (3.99)
Maternal educational level, *n* (%)^‡^	
1. Grammar school 10 years	21 (10.1)
2. Secondary school 12 years	25 (12.0)
3. Vocational training school	19 (9.1)
4. Further education 1–2 years	38 (18.3)
5. Tertiary education 3–4 years (Bachelor level)	75 (36.1)
6. Advanced education (post‐graduate)	28 (13.5)
7. NA	2 (1.0)
Ethnicity, European, *n* (%)^‡^	206 (99)
Smoking during pregnancy (yes), *n* (%)^‡^	13 (6.2)
Parity (multi), *n* (%)^‡^	94 (45.2)
Gestational weight gain (kg)^†^	9.68 (6.28)
**Paternal characteristics**	***n* = 180**
BMI at enrolment (kg/m^2^)^†^	27.26 (4.50)
**Offspring characteristics**	***n* = 208**
Sex, *n* (%)^‡^	
Female	100 (48.1)
Male	108 (51.9)
Gestational age (weeks)^†^	40.11 (1.26)
Birthweight (g)^†^	3707.64 (492.31)
Birth length (cm)^†§^	52.49 (2.18)
Lean mass (%)^†¶^	88.93 (4.47)

^†^Mean (SD). ^‡^Frequencies. ^§^n = 200. ^¶^n = 139. Abbreviations: NA, not available; BMI, body mass index; SD, standard deviation.

**FIGURE 1:**
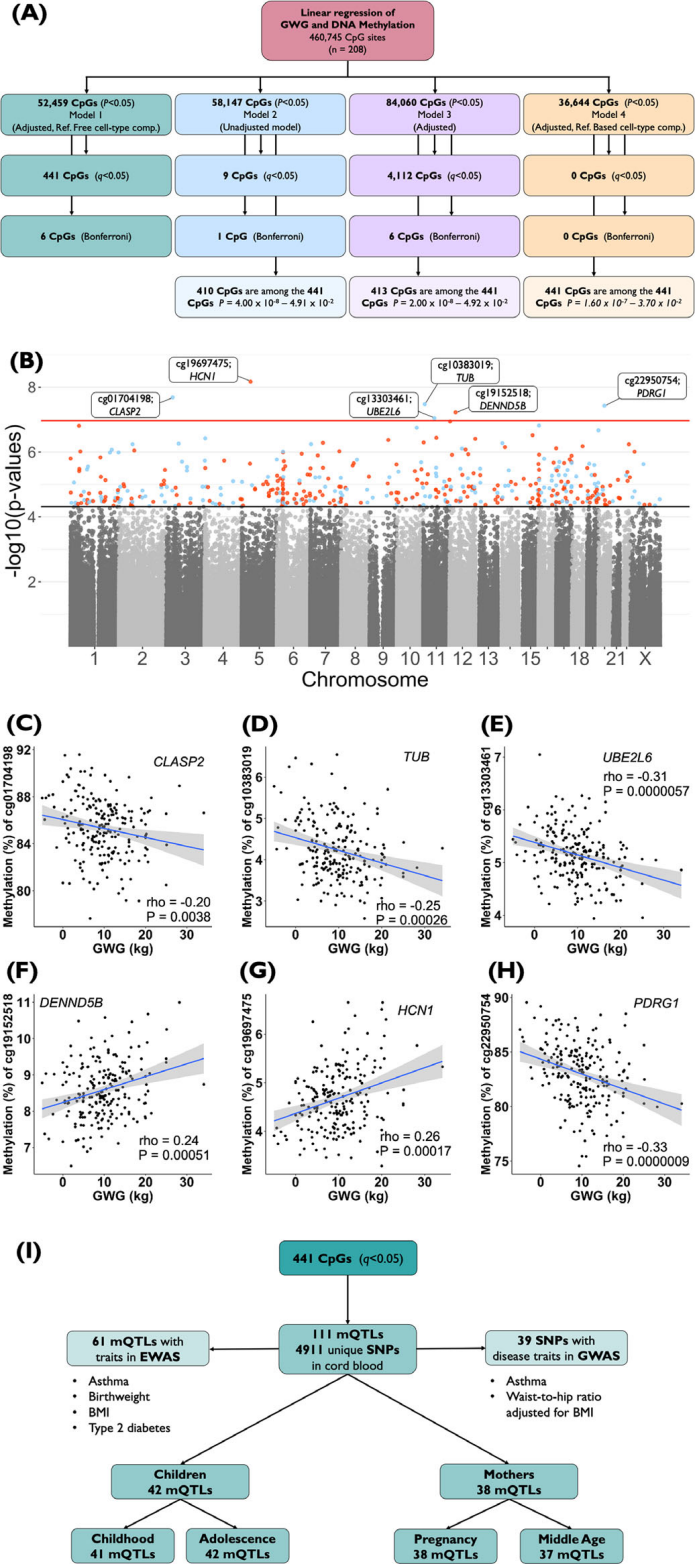
(A) We performed four linear regression models, including different covariates and adjustments for cell‐type composition, investigating the association between gestational weight gain (GWG) and cord blood DNA methylation. Model 1 adjusted for covariates: maternal age (years), pre‐pregnancy body mass index (BMI; kilograms per meter squared), lifestyle intervention (yes/no), gestational age (GA; days), and offspring sex, and adjustment for cell‐type composition using the reference‐free method from Houseman et al., Model 2 unadjusted model, Model 3 adjusted for covariates as above but not for cell‐type composition, Model 4 adjusted for covariates as above and adjustment for cell‐type composition using a reference‐based method. There are different methods adjusting for cell‐type composition (e.g. reference‐based versus reference‐free), which have their pros and cons. Therefore, both methods (Model 1 and 4) were used in this study showing similar results. (B) A Manhattan plot, representing the distribution of methylation sites across the genome, for the association between GWG and DNA methylation in cord blood from the offspring, after adjustment for covariates and cell‐type composition adjustment (Model 1). The bottom (black) line indicates the FDR‐adjusted *P*‐value threshold (*q <* 0.05), of which 441 sites surpassed, and the top (red) line indicates the Bonferroni threshold (1.085199 × 10^−07^, i.e., 0.05/460,745) of which six sites surpassed (based on Model 1). We used both Benjamini‐Hochberg and Bonferroni to correct for multiple testing. This was done because, in epigenome‐wide association studies (EWAS), Bonferroni is known to be too conservative due to correlating DNA methylation values at nearby sites and the non‐variability of several sites on the array. Whereas the potentially more powerful method, the Benjamini‐Hochberg adjustment may produce some false‐positive results. Methylation sites surpassing the FDR threshold (FDR less than 5%, *q <* 0.05) are highlighted in colour; red is hypermethylated, and blue is hypomethylated. Hyper‐/hypomethylation is based on beta coefficients from model 2, an unadjusted model without cell‐type composition adjustment. Data are also presented in Table S1. Spearman correlation plots of the six sites that remained statistically significant after Bonferroni correction (*P* < 1.085199 × 10^−07^) in Model 1; (C) cg01704198 in the gene body of *CLASP2, rho* =−0.20, *P* = 0.0038; (D) cg10383019, in the gene body of *TUB, rho* =−0.25, *P* = 0.00026; (E) cg13303461 in the promoter region of *UBE2L6, rho* =−0.31, *P* = 0.0000057; (F) cg19152518 in the 1st Exon and 5’ untranslated region of *DENND5B, rho* = 0.24, *P* = 0.00051; (G) cg19697475, in the promotor region of *HCN1, rho* = 0.26, *P* = 0.00017; and (H) cg22950754 in the promotor region of *PDRG1, rho* =−0.33, *P* = 0.0000009. (I) Presents previously identified mQTLs of DNA methylation sites in cord blood, which we found associated with GWG. Several of these mQTLs are associated with traits in published GWAS and EWAS, for example, asthma, birthweight, BMI, and type 2 diabetes. Part of the identified mQTLs in cord blood was also identified in children and mothers (whole blood). These data are also presented in Tables S2–S5. Abbreviations: CpGs, DNA methylation sites; DXA, Dual‐energy X‐ray absorptiometry; EWAS, epigenome‐wide association studies; FDR, false discovery rate; GWAS, genome‐wide association studies; GWG, gestational weight gain; mQTL, methylation Quantitative Trait Loci.

We proceeded to study the genetic influence on DNA methylation in cord blood of the 441 GWG‐associated sites using the methylation Quantitative Trait Loci (mQTL) database.^6^ 4911 single nucleotide polymorphisms (SNPs) have been associated with cord blood DNA methylation of 111 of our 441 sites, so‐called mQTLs (Figure 1I and Tables S2–S5). Among these mQTLs, 39 SNPs were associated with disease traits in the genome‐wide association studies catalogue, including asthma (e.g. genes *SIK2* and *WDR36*) and waist‐to‐hip ratio adjusted for BMI (in gene *ATP6V0A2*) (Figure 1I and Table S2).^7^ Moreover, methylation of several of these mQTLs have been linked to BMI (cg12338137 in the gene body of *TNS1*), birthweight (e.g. cg22441770 in the gene body of *CRTC2*, and cg24796852 in *GMFG* promotor), and asthma (e.g. cg21689291 in the gene body of *TMEM106A*) in published EWAS (Tables S2–S5).

Furthermore, we have previously shown that offspring of mothers with obesity are born with higher fat mass^8^ and that carbohydrate intake in late gestation in pregnant women with obesity is positively associated with fat mass in their offspring at birth.^9^ However, when women with obesity underwent lifestyle interventions during pregnancy, the offspring were born with more lean mass than the offspring of women assigned a control intervention.^10^ Therefore, we explored if GWG is also associated with lean mass at birth in 139 offspring. We found a negative correlation between GWG and offspring lean mass at birth (Figure 2A). When performing a linear model adjusting for lifestyle intervention, smoking, GA, and sex, it was estimated that with every kilogram of GWG, lean mass at birth decreased in the offspring by 0.23 ± 0.05 percentage points (95% CI: ‐0.33; ‐0.13). We next analyzed whether GWG was associated with offspring birthweight in 208 offspring and found a positive correlation between GWG and offspring birthweight (Figure 2B), in line with published data^1^. After adjustments, birthweight was estimated to increase by 21.1 ± 5.0 g (95% CI: 11.3; 30.9) for every unit of GWG. Thereafter, we tested whether cord blood methylation of our 441 sites was associated with offspring lean mass and birthweight. DNA methylation at 62 sites was associated with offspring lean mass, while methylation at 21 sites was associated with offspring birthweight (Tables S6 and S7). Methylation of 16 of the sites associated with both offspring anthropometric measurements.

**FIGURE 2:**
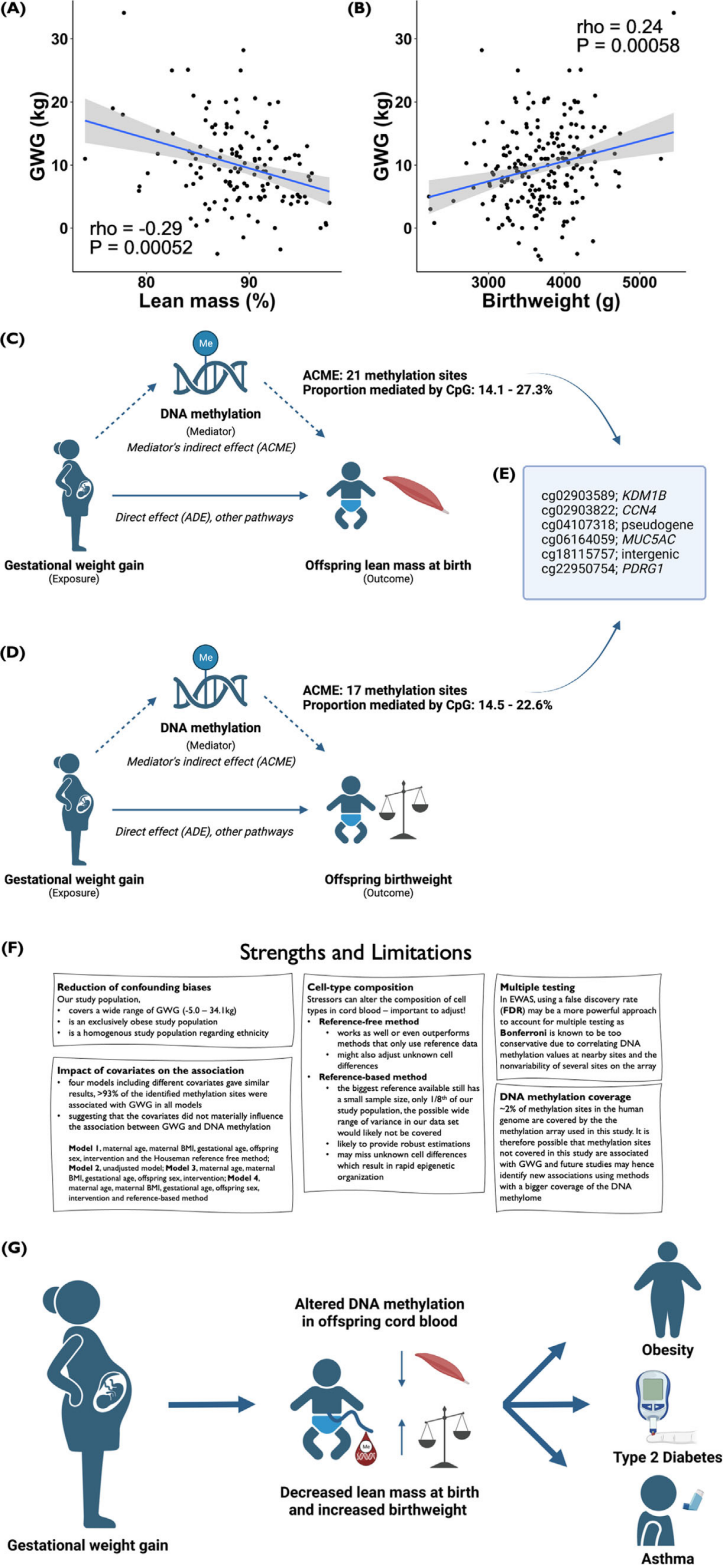
Spearman correlation plots of GWG (kg) and (A) lean mass (%), *rho* =−0.29, *P* = 0.00052 and (B) birthweight (g), *rho* = 0.24, *P* = 0.00058. Causal mediation analyses were performed using the discovered GWG‐associated DNA methylation sites to investigate whether DNA methylation in cord blood of these sites is part of a pathway through which GWG exerts its effects on (C) lean mass and (D) birthweight. Here (C and D), we show the scheme tested by the causal mediation analysis and the potential mechanisms linking GWG and offspring anthropometric measurements. The solid blue arrow represents the effect of GWG on offspring anthropometric measurements that operate directly (ADE) or through a pathway different from the mediator analyzed in the current study (DNA methylation in cord blood). The dotted blue arrows represent a suggested alternative pathway, where an indirect effect (ACME) of GWG on offspring anthropometric measurements is mediated by cord blood DNA methylation. The effect is estimated for each association between GWG (exposure) and lean mass (62 sites) or birthweight (21 sites) (outcome(s)) using the 441 discovered GWG‐associated DNA methylation sites in cord blood. DNA methylation of each respective site was chosen as the mediator. (E) presents the six sites, and their annotated gene (cg02903589, in the gene body of *KDM1B*; cg02903822, in the 5’UTR/1^st^ Exon of *CCN4*; cg04107318, pseudogene; cg06164059, annotated to *MUC5AC*; cg18115757, intergenic; and cg22950754, in the promotor of *PDRG1*), that are suggested to partially mediate the effect of GWG on both offspring lean mass and birthweight. Models regarding lean mass were adjusted for lifestyle intervention, maternal smoking during pregnancy, gestational age, and offspring sex. Models regarding offspring birthweight were adjusted for gestational age and parity. Created with BioRender.com. (F) Presents some observed Strengths and Limitations in this work. (G) Hypothesized pathway linking maternal GWG to intrauterine metabolic programming, mediated by DNA methylation, which in turn may affect anthropometric measurements of importance for the future health of the offspring and finally lead to increased risk of developing obesity, type 2 diabetes, and asthma in the offspring. Created with BioRender.com. Abbreviations: GWG, gestational weight gain; ACME, average causal mediator effect; ADE, average direct effect; rho, Spearman correlation coefficient.

We then performed causal mediation analyses to investigate whether the effect of GWG on the offspring's lean mass and/or birthweight was partially mediated through cord blood DNA methylation of any of the lean mass (62 sites) and/or birthweight (21 sites) associated sites (Figure 2C–E). The mediation analysis breaks down the total effect of exposure (GWG) on the outcome (offspring's lean mass/birthweight) into two parts: first, the indirect effect acting via the mediator of interest (DNA methylation), and second, the direct effect acting directly or via a mediator other than what is under study (Figure 2C,D). We found DNA methylation at 21 and 17 sites to partially mediate the effect of GWG on offspring's lean mass and birthweight, respectively, including methylation sites annotated to *CCN4, KDM1B*, and *MUC5AC* (FDR<5%, Table 2).

**TABLE 2:** Causal mediation analysis on the found gestational weight gain (GWG)‐associated lean mass or birthweight DNA methylation (CpG) sites as mediators and total lean mass (%) or birthweight (g) as the outcome (ACME *q*‐value < 0.05). The causal mediation analysis showed that 1) GWG has an overall effect of β = –0.235 (95% CI: –0.343––0.134) on the offspring's lean mass and of β = 21.087 (95% CI: 9.363–31.862) on birthweight, 2) part of that effect may operate via an indirect path (indirect effect), possibly through DNA methylation, with an average causal mediator effect (ACME) for 21 methylation sites for lean mass and 17 sites for birthweight (*q* < 0.05), 3) another part of that effect goes directly or via mediator(s) other than the 21 and 17 methylation sites, called average direct effect (ADE) and 4) consequently, 14.1%–27.3% of the total effect of GWG on lean mass is suggested to act via these 21 methylation sites, and 14.5%–22.6% of the total effect of GWG on birthweight is suggested to act via the, above mentioned, 17 methylation sites

**Anthropometry**	**CpG site**	**Gene symbol,** **hg38,** **GENCODE** **version 36**	**Gene symbol, Illumina**	**ACME estimate of mediator CpG** **(95% CI)**	**ACME** ***q‐*value**	**ADE estimate (95% CI)**	**Total Effect** **(95% CI)**	**Proportion mediated by CpG** **(95% CI)**
Lean mass	cg02903822	*CCN4*	*WISP1*	−0.047 (−0.092; −0.013)	0.0193	−0.188 (−0.297; −0.086)	−0.235 (−0.343; −0.134)	0.198 (0.055; 0.440)
	cg06735598	*ATP6V0A2*	*ATP6V0A2*	−0.061 (−0.123; −0.018)	0.0193	−0.174 (−0.273; −0.070)	−0.235 (−0.343; −0.134)	0.259 (0.082; 0.588)
	cg12177562	*PNCK;SLC6A8*	*SLC6A8*	−0.042 (−0.082; −0.011)	0.0193	−0.193 (−0.298; −0.098)	−0.235 (−0.343; −0.134)	0.178 (0.052; 0.385)
	cg13692739			−0.053 (−0.097; −0.015)	0.0193	−0.181 (−0.284; −0.087)	−0.235 (−0.343; −0.134)	0.228 (0.067; 0.448)
	cg15715256	*CCDC152*	*CCDC152*	−0.051 (−0.102; −0.017)	0.0193	−0.184 (−0.286; −0.070)	−0.235 (−0.343; −0.134)	0.217 (0.078; 0.535)
	cg22950754	*PDRG1*	*PDRG1*	−0.063 (−0.114; −0.020)	0.0193	−0.172 (−0.284; −0.063)	−0.235 (−0.343; −0.134)	0.268 (0.084; 0.610)
	cg15848714	*KIAA1549L*		−0.052 (−0.110; −0.011)	0.0290	−0.183 (−0.286; −0.077)	−0.235 (−0.343; −0.134)	0.223 (0.055; 0.515)
	cg22776687	*AL513210.1*		−0.052 (−0.103; −0.015)	0.0290	−0.183 (−0.281; −0.082)	−0.235 (−0.343; −0.134)	0.223 (0.066; 0.473)
	cg02429905	*AL662884.4;PRRT1*	*PRRT1;PPT2*	−0.046 (−0.096; −0.010)	0.0316	−0.189 (−0.293; −0.090)	−0.235 (−0.343; −0.134)	0.196 (0.049; 0.417)
	cg04980478			−0.046 (−0.085; −0.013)	0.0316	−0.189 (−0.293; −0.091)	−0.235 (−0.343; −0.134)	0.197 (0.058; 0.393)
	cg18115757			−0.035 (−0.082; −0.007)	0.0316	−0.200 (−0.309; −0.095)	−0.235 (−0.343; −0.134)	0.151 (0.031; 0.396)
	cg04107318	*COTL1P1;KRT17P4*		−0.051 (−0.094; −0.014)	0.0357	−0.184 (−0.290; −0.087)	−0.235 (−0.343; −0.134)	0.217 (0.067; 0.467)
	cg05626094	*TSC2*	*TSC2*	−0.049 (−0.099; −0.015)	0.0357	−0.186 (−0.292; −0.081)	−0.235 (−0.343; −0.134)	0.209 (0.069; 0.496)
	cg06164059	*MUC5AC*		−0.040 (−0.081; −0.008)	0.0387	−0.195 (−0.306; −0.096)	−0.235 (−0.343; −0.134)	0.171 (0.037; 0.381)
	cg24397737			−0.041 (−0.083; −0.009)	0.0387	−0.194 (−0.298; −0.096)	−0.235 (−0.343; −0.134)	0.175 (0.039; 0.378)
	cg08682866	*COX10‐AS1*		−0.059 (−0.117; −0.011)	0.0435	−0.176 (−0.290; −0.063)	−0.235 (−0.343; −0.134)	0.251 (0.044; 0.574)
	cg13706079	*SAMD4A*	*SAMD4A*	−0.039 (−0.092; −0.007)	0.0442	−0.196 (−0.309; −0.079)	−0.235 (−0.343; −0.134)	0.168 (0.026; 0.499)
	cg22022716	*BNIP3L*		−0.036 (−0.082; −0.004)	0.0442	−0.199 (−0.302; −0.100)	−0.235 (−0.343; −0.134)	0.151 (0.021; 0.387)
	cg23926700	*POGLUT3*	*KDELC2*	−0.045 (−0.094; −0.010)	0.0442	−0.189 (−0.303; −0.093)	−0.235 (−0.343; −0.134)	0.194 (0.043; 0.434)
	cg24413369	*AC010969.3;GRHL1*	*GRHL1*	−0.038 (−0.087; −0.004)	0.0442	−0.196 (−0.298; −0.099)	−0.235 (−0.343; −0.134)	0.164 (0.018; 0.388)
	cg24413842	*GIMAP7;STRADBP1*	*GIMAP7*	−0.030 (−0.064; −0.004)	0.0442	−0.205 (−0.305; −0.108)	−0.235 (−0.343; −0.134)	0.128 (0.015; 0.297)
	cg02903589	*KDM1B*	*KDM1B*	−0.038 (−0.079; −0.005)	0.0454	−0.197 (−0.291; −0.098)	−0.235 (−0.343; −0.134)	0.163 (0.025; 0.376)
	cg16908944	*AP002518.1*		−0.033 (−0.071; −0.005)	0.0454	−0.202 (−0.310; −0.099)	−0.235 (−0.343; −0.134)	0.139 (0.023; 0.347)
	cg20210689	*INPP5A*	*INPP5A*	−0.031 (−0.066; −0.004)	0.0483	−0.204 (−0.310; −0.100)	−0.235 (−0.343; −0.134)	0.132 (0.018; 0.336)
	cg08301597	*NDUFA12*	*NDUFA12*	−0.046 (−0.094; −0.007)	0.0491	−0.189 (−0.303; −0.076)	−0.235 (−0.343; −0.134)	0.196 (0.031; 0.496)
	cg23164850	*FSCN2*	*FSCN2*	−0.038 (−0.080; −0.005)	0.0491	−0.197 (−0.304; −0.093)	−0.235 (−0.343; −0.134)	0.162 (0.020; 0.395)
	cg03899643	*AC093423.3;LRRC8C*		−0.034 (−0.072; −0.004)	0.0497	−0.201 (−0.296; −0.107)	−0.235 (−0.343; −0.134)	0.146 (0.019; 0.304)
	cg07764334	*AC011092.2;SBF2*	*SBF2*	−0.031 (−0.073; −0.003)	0.0497	−0.204 (−0.309; −0.104)	−0.235 (−0.343; −0.134)	0.131 (0.014; 0.330)
Birthweight	cg18115757			4.764 (1.699; 9.521)	<0.001	16.323 (3.741; 27.365)	21.087 (9.363; 31.862)	0.226 (0.077; 0.643)
	cg02903822	*CCN4*	*WISP1*	4.198 (1.233; 8.178)	0.0315	16.889 (5.059; 27.542)	21.087 (9.363; 31.862)	0.199 (0.057; 0.520)
	cg03015498			3.912 (0.697; 8.527)	0.0315	17.175 (6.093; 27.767)	21.087 (9.363; 31.862)	0.185 (0.037; 0.454)
	cg06691250			3.805 (0.759; 8.043)	0.0315	17.282 (6.112; 27.558)	21.087 (9.363; 31.862)	0.180 (0.037; 0.461)
	cg04107318	*COTL1P1;KRT17P4*		3.883 (0.899; 7.463)	0.0350	17.204 (5.582; 27.884)	21.087 (9.363; 31.862)	0.184 (0.047; 0.482)
	cg06164059	*MUC5AC*		3.386 (0.582; 7.136)	0.0350	17.701 (6.493; 28.443)	21.087 (9.363; 31.862)	0.161 (0.027; 0.424)
	cg00869796	*AC063944.1*		3.180 (0.455; 6.587)	0.0360	17.907 (5.654; 29.069)	21.087 (9.363; 31.862)	0.151 (0.024; 0.423)
	cg01715572	*ARMC9*	*ARMC9*	3.919 (0.569; 7.764)	0.0367	17.168 (6.631; 27.663)	21.087 (9.363; 31.862)	0.186 (0.036; 0.445)
	cg02903589	*KDM1B*	*KDM1B*	3.051 (0.641; 6.345)	0.0420	18.036 (6.174; 29.420)	21.087 (9.363; 31.862)	0.145 (0.027; 0.404)
	cg03899643	*AC093423.3;LRRC8C*		3.277 (0.446; 7.046)	0.0420	17.810 (6.538; 28.048)	21.087 (9.363; 31.862)	0.155 (0.023; 0.420)
	cg11559571	*PCSK6*		3.143 (0.491; 5.953)	0.0420	17.944 (6.334; 28.594)	21.087 (9.363; 31.862)	0.149 (0.020; 0.390)
	cg12184854	*TP63*	*TP63*	3.408 (0.257; 7.368)	0.0420	17.678 (6.035; 28.248)	21.087 (9.363; 31.862)	0.162 (0.014; 0.436)
	cg13706079	*SAMD4A*	*SAMD4A*	3.180 (0.356; 7.367)	0.0420	17.907 (6.072; 28.262)	21.087 (9.363; 31.862)	0.151 (0.022; 0.415)
	cg26446816	*AGAP1*	*AGAP1*	3.608 (0.410; 6.912)	0.0420	17.479 (6.016; 27.682)	21.087 (9.363; 31.862)	0.171 (0.024; 0.402)
	cg04958872	*WNT8B*	*WNT8B*	3.709 (0.248; 7.600)	0.0469	17.378 (5.257; 27.569)	21.087 (9.363; 31.862)	0.176 (0.016; 0.485)
	cg09257020	*LINC02781*		3.121 (0.170; 6.966)	0.0469	17.966 (6.755; 28.466)	21.087 (9.363; 31.862)	0.148 (0.012; 0.394)
	cg22950754	*PDRG1*	*PDRG1*	3.386 (0.175; 7.700)	0.0469	17.701 (4.840; 28.923)	21.087 (9.363; 31.862)	0.161 (0.011; 0.515)

Lean mass models are adjusted for lifestyle intervention, maternal smoking during pregnancy, GA (in days), and offspring sex, and based on 139 participants. Birthweight models are adjusted for GA (in days) and parity.

Abbreviations: ACME, average causal mediator effect; ADE, average direct effect; CpG site, DNA methylation site; GA, gestational age.

In this study, we found for the first time associations between GWG in pregnant women with obesity and differential DNA methylation at individual sites in offspring's cord blood. Several identified epigenetic alterations were also associated with the offspring's lean mass and birthweight. Notably, we found that DNA methylation at 21 and 17 sites partially mediates the effect of GWG on lean mass and birthweight in the offspring, respectively. Six methylation sites are proposed to partially mediate the effect of GWG on both lean mass and birthweight. One site resides in *KDM1B*, encoding a histone demethylase that regulates histone lysine methylation.^11^ Additionally, in the present study, GWG was negatively associated with offspring's lean mass and positively associated with offspring birthweight, in line with previous work.^1^, ^12^ Interestingly, one in four of the found GWG‐associated cord blood methylation sites has also been associated with genetic variation, so‐called mQTLs, and traits such as asthma, birthweight, BMI, and type 2 diabetes based on previous EWAS. Hence, our novel data provide evidence that GWG in pregnant women with obesity impacts the methylome in offspring cord blood and on anthropometric measurements with probable importance for the health of the offspring (Figure 2G). Together these data imply that reducing GWG in a population with obesity is relevant to reduce potential long‐term health effects in the offspring.

In conclusion, this study provides evidence that GWG in pregnant women with obesity is associated with cord blood DNA methylation of sites previously linked to BMI, type 2 diabetes, and asthma. We also demonstrate that anthropometric measurements of importance for the future health of the offspring (i.e. lean mass and birthweight) are associated with GWG. This further supports that reducing GWG in women with obesity may be of value for the offspring's future health. These results also stress the importance of the intrauterine environment in humans and its ability to program the methylome, potentially affecting the offspring's metabolism.

## Supporting information

Supporting information

Supporting information

Supporting information

Supporting information
