# Supplementary material for: Gestational weight gain in pregnant women with obesity is associated with cord blood DNA methylation, which partially mediates offspring anthropometrics
Source: Clin Transl Med. 2023 Mar 16;13(3):e1215. doi: 10.1002/ctm2.1215 (PMC10019770; doi:10.1002/ctm2.1215)
Supplement: Supplementary file 1 — Supporting information [file CTM2-13-e1215-s002.docx]

**Supporting Information**

For "Gestational weight gain in pregnant women with obesity is associated with cord blood DNA methylation, which partially mediates offspring anthropometrics"

by Josefine Jönsson, Kristina M. Renault, Alexander Perfilyev, Allan Vaag, Emma Malchau Carlsen, Kirsten Nørgaard, Paul W. Franks, and Charlotte Ling

**Supporting Document:**

Containing **Methods** and **Article Information** (Word)

**Supporting Figure:**

**Figure S1.** Flow diagram (Tiff)

**Supporting Tables:**

**Table S1:** GWG-associated DNA methylation sites in cord blood, based on FDR<5% (*q*-value) (TOP study) (Excel)

**Table S2:** mQTLs in cord blood (mQTL database) found among the GWG-associated methylation sites in cord blood (TOP study), combined with public GWAS data (Excel)

**Table S3:** mQTLs in cord blood and peripheral blood in children (mQTL database) found among the GWG-associated methylation sites in cord blood (TOP study), combined with public GWAS data (Excel)

**Table S4:** mQTLs in cord blood and peripheral blood in mothers (mQTL database) found among the GWG-associated methylation sites in cord blood (TOP study), combined with public GWAS data (Excel)

**Table S5:** mQTLs in cord blood (mQTL database) found among the GWG-associated methylation sites in cord blood (TOP study) and overlap with the epigenome-wide association studies (EWAS) database (Excel)

**Table S6:** Association models of total lean mass (%) at birth and the found GWG-associated methylation sites in cord blood, based on FDR<5% (*q*-value) (TOP study) (linear regression models adjusted for lifestyle intervention, maternal smoking during pregnancy, gestational age, and offspring sex) (Excel)

**Table S7:** Association models of birthweight (g) and the found GWG-associated methylation sites found in cord blood, based on FDR<5% (*q*-value) (TOP study) (linear regression models adjusted for offspring gestational age and parity (multi/single)) (Excel)
