# Supplementary material for: Gestational weight gain in pregnant women with obesity is associated with cord blood DNA methylation, which partially mediates offspring anthropometrics
Source: Clin Transl Med. 2023 Mar 16;13(3):e1215. doi: 10.1002/ctm2.1215 (PMC10019770; doi:10.1002/ctm2.1215)
Supplement: Supplementary file 2 — Supporting information [file CTM2-13-e1215-s003.docx]

**Gestational weight gain in pregnant women with obesity is associated with cord blood DNA methylation, which partially mediates offspring anthropometrics**

Josefine Jönsson^1^, Kristina M. Renault^2,3^, Alexander Perfilyev^1^, Allan Vaag^5^, Emma Malchau Carlsen^6,7^, Kirsten Nørgaard^5^, Paul W. Franks^4^, and Charlotte Ling^1^

^1^ Epigenetics and Diabetes Unit, Department of Clinical Sciences, Lund University Diabetes Centre, Lund University, Scania University Hospital, Malmö, Sweden

^2^ Department of Obstetrics and Gynecology, Hvidovre Hospital, University of Copenhagen, Copenhagen, Denmark.

^3^ Department of Obstetrics, Juliane Marie Centret, Rigshospitalet, University of Copenhagen, Copenhagen, Denmark.

^4^ Genetic and Molecular Epidemiology Unit, Department of Clinical Sciences, Lund University Diabetes Centre, Lund University, Malmö, Sweden

^5^ Steno Diabetes Center Copenhagen, Gentofte, Denmark

^6^ Department of Nutrition, Exercise and Sports, Faculty of Science, University of Copenhagen, Frederiksberg, Denmark

^7^ Department of Pediatrics, Copenhagen University Hospital Hvidovre, Hvidovre, Denmark.

Corresponding authors: Josefine Jönsson and Charlotte Ling, Lund University Epigenetics and Diabetes, Clinical Research Centre 91:12, Box 50332, 202 13 Malmö, Sweden.

Phone: +46 40 39 12 11; e-mail: josefin.jonsson@med.lu.se and charlotte.ling@med.lu.se

### **METHODS**

#### **Research Design and Clinical Data of the TOP-Study**

The Ethics Committee for the Capital Region of Denmark approved the TOP study (January 2009, H-D-2008–119; Hillerød, Denmark) and is registered at ClinicalTrials.gov (NCT01345149). Written informed consent was obtained from all participants before enrolment. The TOP study is a triple-arm randomized controlled trial of 425 pregnant women with obesity at the time of enrolment. Due to participants withdrawing from the study, moving from the region, and having miscarriages, 389 of the 425 enrolled women completed the study. The trial includes two interventions: physical activity (assessed with a pedometer) and dietary advice (PA + D) and physical activity alone (PA), and a control arm receiving standard of care (C) (Figure S1). The trial’s primary endpoint was GWG (1).

Maternal age, pre-pregnancy BMI, and parity were recorded at enrolment (weeks 11–14). GWG was defined as the difference between the measured weight during weeks 36 and 37 and the self-reported pre-pregnancy weight. The self-reported pre-pregnancy weight was measured and validated at the first visit in weeks 11-14. If there was a considerable difference between the self-reported and measured weight, the measured weight was used. Detailed information regarding the conduction of the trial, clinical measurements, and enrolment is reported elsewhere (1-3). Smoking habits during pregnancy were obtained using medical records.

The body composition of the offspring was measured using dual-energy X-ray absorptiometry scans within 48 h of birth. Offspring measurements used in the current analysis were lean mass at birth and birthweight. Body composition assessment in the offspring and data collection have been described elsewhere (4, 5).

At birth, cord blood was collected from the umbilical vein of the clamped umbilical cord. Blood samples were frozen (-80°C) and stored in a biobank at Copenhagen University Hospital Hvidovre. Samples of whole-cord blood were available for 232 participants (Figure S1).

**DNA methylation Analyses**

DNA was extracted from cord blood using the QIAamp 96 DNA Blood Kit and concentration and purity were determined using NanoDrop (NanoDrop Technologies, Inc.). Eight samples lacked sufficient DNA concentrations. The remaining samples were bisulfite-converted with the EZ-96 DNA methylation kit (Zymo Research Corporation, Irvine, CA, USA) of which six samples failed bisulfite conversion. DNA methylation in 218 samples was analyzed with Illumina Infinium HumanMethylation450 BeadChips (Illumina, San Diego, CA, USA), covering 485,577 sites (6). Imaging of the Infinium Human-Methylation450K BeadChips was performed by Illumina iScan. Three samples lacked data on GWG and were removed. Preprocessing of data was performed using R (7) (version 4.0.2), and Bioconductor packages; methylumi (8), lumi (9), sva (10), minfi (11), and limma (12). Methylation values were converted to β-values (percentage) using the formula; β = intensity of the Methylated allele (M)/(intensity of the Unmethylated allele [U] + intensity of the Methylated allele [M] + 100). A total of 416 probes annotated to the Y chromosome, 65 rs-probes, 3,091 ch-probes, 14,466 probes based on cross-reactivity (13), 5,216 polymorphic probes with a minor allele frequency of 0.1 (14), and 1,578 probes with average detection *P*-value ≥0.01 were filtered out. Methylation data were obtained for 460,745 probes. M-values were calculated and used for further analyses (M = log2(β /[1- β])) (15). Quantile normalization and background correction were performed, and the beta-Mixture Quantile normalization method (16) was applied. ComBat was used to correct for batch effects (17, 18). To ensure that between-array batch effects were removed principal component analyses (PCA) before and after applying ComBat were performed. An additional seven participants were excluded, due to clustering to the wrong sex in the PCA. Subsequently, DNA methylation data were available for 208 participants, 76, 59, and 73, in PA + D, PA, and C, respectively (Figure S1 and Table 1). Additional gene annotation was performed using hg38, GENCODE version 36.

**Statistical analyses**

To test if cord blood DNA methylation is associated with GWG independent of treatment allocation, a linear regression model adjusted for covariates was used: maternal age (years), pre-pregnancy BMI (kilograms per meter squared), lifestyle intervention (yes/no), gestational age at birth (GA; days), and offspring sex. Cell composition was adjusted using the reference-free method from Houseman et al. (19) (Model 1, Figure 1A, Table S1). To estimate the covariates’ influence on the association, we additionally examined three models: Model 2, an unadjusted model without cell composition adjustment; Model 3, adjusted for covariates as above but without cell composition adjustment; and Model 4 adjusted for covariates as above and cell composition adjustment using a reference-based method (20) (Figure 1A, Table S1). We estimated the absolute cell proportions from our cord blood DNA methylation data using the filtered and combined reference dataset “FlowSorted.CordBloodCombined.450k”, available via Bioconductor (21). The estimate-CellCounts2() function in the FlowSorted.Blood.EPIC R package (22) was used, where we specified all seven cell types (CD8+ T lymphocytes, CD4+ T lymphocytes, NK cells, B cells, monocytes, granulocytes, and nucleated red blood cells), used IDOL probe selection for deconvolution and noob preprocessing. Moreover, to correct for multiple testing we used both Benjamini-Hochberg and Bonferroni. In EWAS, Bonferroni is known to be too conservative because of correlating DNA methylation values at nearby sites and the nonvariability of several sites on the array (23, 24). A potentially more powerful method is Benjamini-Hochberg adjustment, it may however produce a few false-positive results. As pregnant women with obesity more often exceed the Institute of Medicine recommendations regarding GWG compared to normal-weight women (25) we investigated whether any of our found GWG-associated methylation sites have previously been linked to pre-pregnancy BMI (26-28). To further validate our results, using Model 1, we randomly split our cohort into a discovery and validation cohort (60:40) and examined the overlap between these two analyses and the initial analysis including the full cohort.

Linear regression models were used to assess whether cord blood DNA methylation at sites found to associate with GWG is associated with offspring lean mass at birth and with birthweight. Covariates with putative biological impact on DNA methylation and lean mass or birthweight, significant at *P* < 0.25 in univariate analyses, were incorporated in the final regression models (29). For both anthropometries (lean mass and birthweight) the following variables were considered: lifestyle intervention (yes/no), maternal pre-pregnancy BMI, maternal smoking during pregnancy (yes/no), GA at birth, parity (single/multi), and offspring sex. For lean mass, included variables were lifestyle intervention, maternal smoking during pregnancy, GA, and offspring sex. Regarding offspring birthweight, the adjusted regression model included GA and parity. In these linear regression models, lean mass or birthweight was the dependent variable and DNA methylation of the respective site was the independent variable. Normalized methylation β-values were used for the linear regressions regarding offspring anthropometrics. Variance-inflation factors (VIF) were calculated to check for multicollinearity in the performed regression models. No covariates were excluded due to multicollinearity (VIF≤1.07). All statistical analyses were performed using the software R (7) (versions 4.0.2, and 4.1.1) and RStudio (30). Unless stated otherwise, data are presented as Estimated β ± Standard Error, and models were corrected for multiple testing using false discovery rate (FDR) analysis (Benjamini-Hochberg) in which FDR <5% (*q*<0.05) was used as a cut-off. Bonferroni multiple comparison post hoc corrections are also presented when analyzing whether GWG is associated with cord blood DNA methylation.

**Genetic Influence**

To study the genetic influences on DNA methylation in cord blood of the 441 methylation sites found to associate with GWG we used the mQTL (methylation Quantitative Trait Loci) database (31) (accessed on 2022-02-07). Using the genome-wide association studies (GWAS) catalog (accessed on 2022-02-07) (32) and the epigenome-wide association studies (EWAS) Atlas (33) (accessed on 2022-02-08) we investigated if the found SNPs were associated with disease traits.

**Causal Mediation Analysis**

Using the discovered GWG-associated DNA methylation sites which were also shown to be associated with either lean mass (62 sites) or birthweight (21 sites), we further investigated whether DNA methylation is part of a pathway through which GWG exerts its effects on lean mass (Figure 2C) and/or birthweight (Figure 2D). This was done using a nonparametric causal mediation analysis and the R ‘mediation’ package with default settings (34). The effect is estimated for each association between GWG (treatment) and lean mass or birthweight (outcome(s)) with the discovered GWG-associated DNA methylation sites. DNA methylation of each respective site was chosen as the mediator. Models regarding lean mass were adjusted for lifestyle intervention, maternal smoking during pregnancy, GA, and offspring sex. Models regarding offspring birthweight were adjusted for GA and parity. Both models were corrected for multiple testing using FDR <5% (q<0.05).

**Data availability**

DNA methylation data from cord blood of the TOP study (accession number LUDC2020.08.14) are deposited in the Lund University Diabetes Centre repository (https://www.ludc.lu.se/resources/repository) and are available upon request.

### **ARTICLE INFORMATION**

**Acknowledgments.** The authors would like to thank all study participants of the TOP study, researchers, and clinicians who contributed to this work. The authors also thank Marlena Maziarz, Department of Clinical Sciences, Lund University/CRC, Skåne University Hospital SUS, Malmö, Sweden, for her biostatistics guidance and expertise.

**Funding.** Sygekassernes Helsefond, Brødrene Hartmann Fonden, Hvidovre Hospitals Forskningsfond and The Danish Council for Strategic Research supported the TOP-study. The work performed by P.W.F. was supported by grants from the EFSD, Swedish Research Council, Swedish Heart-Lung Foundation, and the European Research Council (CoG-2015_681742_NASCENT) and Novo Nordisk Foundation. The work performed by J.J., S.G-C., A.P., and C.L. was supported by grants from the Novo Nordisk Foundation, Swedish Research Council, Region Skåne (ALF), ERC-Co Grant (PAINTBOX, No 725840), H2020-Marie Skłodowska-Curie grant agreement No 706081 (EpiHope), Hjärt-Lungfonden, Exodiab, Swedish Foundation for Strategic Research for IRC15-0067 and Swedish Diabetes Foundation. All researchers from LUDC were supported by a research center grant from The Swedish Foundation of Strategic Research.

**Duality of Interest.** No potential conflicts of interest relevant to this article were reported.

**Author Contributions.** J.J. analyzed data, performed statistical analyses, and drafted and revised the manuscript. K.M.R., A.V., P.F.W., and C.L. designed and planned the current study and participated in drafting the manuscript. K.M.R and K.N. designed and planned the TOP study. K.M.R. and E.M.C. conducted the TOP study and collected data. A.P. performed the DNA methylation analyses. P.W.F. and C.L. supervised the analyses.

All authors reviewed and provided critical comments on the manuscript.

J.J., K.M.R., P.W.F., and C.L. are the guarantors of this work and, as such, had full access to all the data in the study and take responsibility for the integrity of the data and the accuracy of the data analysis.

**REFERENCES**

1. Renault KM, Norgaard K, Nilas L, Carlsen EM, Cortes D, Pryds O, et al. The Treatment of Obese Pregnant Women (TOP) study: a randomized controlled trial of the effect of physical activity intervention assessed by pedometer with or without dietary intervention in obese pregnant women. Am J Obstet Gynecol. 2014;210(2):134.e1-9.

2. Jönsson J, Renault KM, García-Calzón S, Perfilyev A, Estampador AC, Nørgaard K, et al. Lifestyle Intervention in Pregnant Women With Obesity Impacts Cord Blood DNA Methylation, Which Associates With Body Composition in the Offspring. Diabetes. 2021;70(4):854-66.

3. Renault KM, Carlsen EM, Norgaard K, Nilas L, Pryds O, Secher NJ, et al. Intake of Sweets, Snacks and Soft Drinks Predicts Weight Gain in Obese Pregnant Women: Detailed Analysis of the Results of a Randomised Controlled Trial. PLoS One. 2015;10(7):e0133041.

4. Carlsen EM, Renault KM, Norgaard K, Nilas L, Jensen JE, Hyldstrup L, et al. Newborn regional body composition is influenced by maternal obesity, gestational weight gain and the birthweight standard score. Acta Paediatr. 2014;103(9):939-45.

5. Ejlerskov KT, Christensen LB, Ritz C, Jensen SM, Molgaard C, Michaelsen KF. The impact of early growth patterns and infant feeding on body composition at 3 years of age. Br J Nutr. 2015;114(2):316-27.

6. Bibikova M, Barnes B, Tsan C, Ho V, Klotzle B, Le JM, et al. High density DNA methylation array with single CpG site resolution. Genomics. 2011;98(4):288-95.

7. R Core Team (2021). R: A language and environment for statistical computing. R Foundation for Statistical Computing, Vienna, Austria. URL https://www.R-project.org/.

8. Davis S DP, Bilke S, Triche, Jr. T, Bootwalla M. methylumi: Handle Illumina methylation data. R package version 2.28.0. 2018.

9. Du P, Kibbe WA, Lin SM. lumi: a pipeline for processing Illumina microarray. Bioinformatics. 2008;24(13):1547-8.

10. Leek JT JW, Parker HS, Fertig EJ, Jaffe AE, Zhang Y, Storey JD, Torres LC. sva: Surrogate Variable Analysis. p. 2021. R package version 3.36.0.

11. Aryee MJ, Jaffe AE, Corrada-Bravo H, Ladd-Acosta C, Feinberg AP, Hansen KD, et al. Minfi: a flexible and comprehensive Bioconductor package for the analysis of Infinium DNA methylation microarrays. Bioinformatics. 2014;30(10):1363-9.

12. Ritchie ME, Phipson B, Wu D, Hu Y, Law CW, Shi W, et al. limma powers differential expression analyses for RNA-sequencing and microarray studies. Nucleic Acids Research. 2015;43(7):e47-e.

13. Chen Y-a, Lemire M, Choufani S, Butcher DT, Grafodatskaya D, Zanke BW, et al. Discovery of cross-reactive probes and polymorphic CpGs in the Illumina Infinium HumanMethylation450 microarray. Epigenetics. 2013;8(2):203-9.

14. Prince JA, Feuk L, Howell WM, Jobs M, Emahazion T, Blennow K, et al. Robust and accurate single nucleotide polymorphism genotyping by dynamic allele-specific hybridization (DASH): design criteria and assay validation. Genome Res. 2001;11(1):152-62.

15. Du P, Zhang X, Huang CC, Jafari N, Kibbe WA, Hou L, et al. Comparison of Beta-value and M-value methods for quantifying methylation levels by microarray analysis. BMC Bioinformatics. 2010;11:587.

16. Teschendorff AE, Marabita F, Lechner M, Bartlett T, Tegner J, Gomez-Cabrero D, et al. A beta-mixture quantile normalization method for correcting probe design bias in Illumina Infinium 450 k DNA methylation data. Bioinformatics. 2013;29(2):189-96.

17. Leek JT, Johnson WE, Parker HS, Jaffe AE, Storey JD. The sva package for removing batch effects and other unwanted variation in high-throughput experiments. Bioinformatics (Oxford, England). 2012;28(6):882-3.

18. Johnson WE, Li C, Rabinovic A. Adjusting batch effects in microarray expression data using empirical Bayes methods. Biostatistics. 2007;8(1):118-27.

19. Houseman EA, Molitor J, Marsit CJ. Reference-free cell mixture adjustments in analysis of DNA methylation data. Bioinformatics. 2014;30(10):1431-9.

20. Gervin K, Salas LA, Bakulski KM, van Zelm MC, Koestler DC, Wiencke JK, et al. Systematic evaluation and validation of reference and library selection methods for deconvolution of cord blood DNA methylation data. Clinical Epigenetics. 2019;11(1):125.

21. Salas LA, Gervin K, Jones MC (2021). FlowSorted.CordBloodCombined.450k: Illumina 450k/EPIC data on FACS and MACS umbilical blood cells. R package version 1.4.1, https://github.com/immunomethylomics/FlowSorted.CordBloodCombined.450k.

22. Salas LA, Koestler DC, Butler RA, Hansen HM, Wiencke JK, Kelsey KT, et al. An optimized library for reference-based deconvolution of whole-blood biospecimens assayed using the Illumina HumanMethylationEPIC BeadArray. Genome Biol. 2018;19(1):64.

23. Mansell G, Gorrie-Stone TJ, Bao Y, Kumari M, Schalkwyk LS, Mill J, et al. Guidance for DNA methylation studies: statistical insights from the Illumina EPIC array. BMC Genomics. 2019;20(1):366.

24. Saffari A, Silver MJ, Zavattari P, Moi L, Columbano A, Meaburn EL, et al. Estimation of a significance threshold for epigenome-wide association studies. Genet Epidemiol. 2018;42(1):20-33.

25. Kaar JL, Crume T, Brinton JT, Bischoff KJ, McDuffie R, Dabelea D. Maternal Obesity, Gestational Weight Gain, and Offspring Adiposity: The Exploring Perinatal Outcomes among Children Study. The Journal of Pediatrics. 2014;165(3):509-15.

26. Martin CL, Jima D, Sharp GC, McCullough LE, Park SS, Gowdy KM, et al. Maternal pre-pregnancy obesity, offspring cord blood DNA methylation, and offspring cardiometabolic health in early childhood: an epigenome-wide association study. Epigenetics. 2019;14(4):325-40.

27. Sharp GC, Lawlor DA, Richmond RC, Fraser A, Simpkin A, Suderman M, et al. Maternal pre-pregnancy BMI and gestational weight gain, offspring DNA methylation and later offspring adiposity: findings from the Avon Longitudinal Study of Parents and Children. Int J Epidemiol. 2015;44(4):1288-304.

28. Sharp GC, Salas LA, Monnereau C, Allard C, Yousefi P, Everson TM, et al. Maternal BMI at the start of pregnancy and offspring epigenome-wide DNA methylation: findings from the pregnancy and childhood epigenetics (PACE) consortium. Hum Mol Genet. 2017;26(20):4067-85.

29. Bursac Z, Gauss CH, Williams DK, Hosmer DW. Purposeful selection of variables in logistic regression. Source Code for Biology and Medicine. 2008;3(1):17.

30. RStudio Team (2020). RStudio: Integrated Development for R. RStudio, PBC, Boston, MA. URL http://www.rstudio.com/.

31. Gaunt TR, Shihab HA, Hemani G, Min JL, Woodward G, Lyttleton O, et al. Systematic identification of genetic influences on methylation across the human life course. Genome Biol. 2016;17:61.

32. Buniello A, MacArthur JAL, Cerezo M, Harris LW, Hayhurst J, Malangone C, et al. The NHGRI-EBI GWAS Catalog of published genome-wide association studies, targeted arrays and summary statistics 2019. Nucleic Acids Res. 2019;47(D1):D1005-d12.

33. Li M, Zou D, Li Z, Gao R, Sang J, Zhang Y, et al. EWAS Atlas: a curated knowledgebase of epigenome-wide association studies. Nucleic Acids Res. 2019;47(D1):D983-d8.

34. Tingley D, Yamamoto T, Hirose K, Keele L, Imai K. mediation: R Package for Causal Mediation Analysis. 2014. 2014;59(5):38.
