# Supplementary figures and images for: Gestational weight gain in pregnant women with obesity is associated with cord blood DNA methylation, which partially mediates offspring anthropometrics
Source: Clin Transl Med. 2023 Mar 16;13(3):e1215. doi: 10.1002/ctm2.1215 (PMC10019770; doi:10.1002/ctm2.1215)

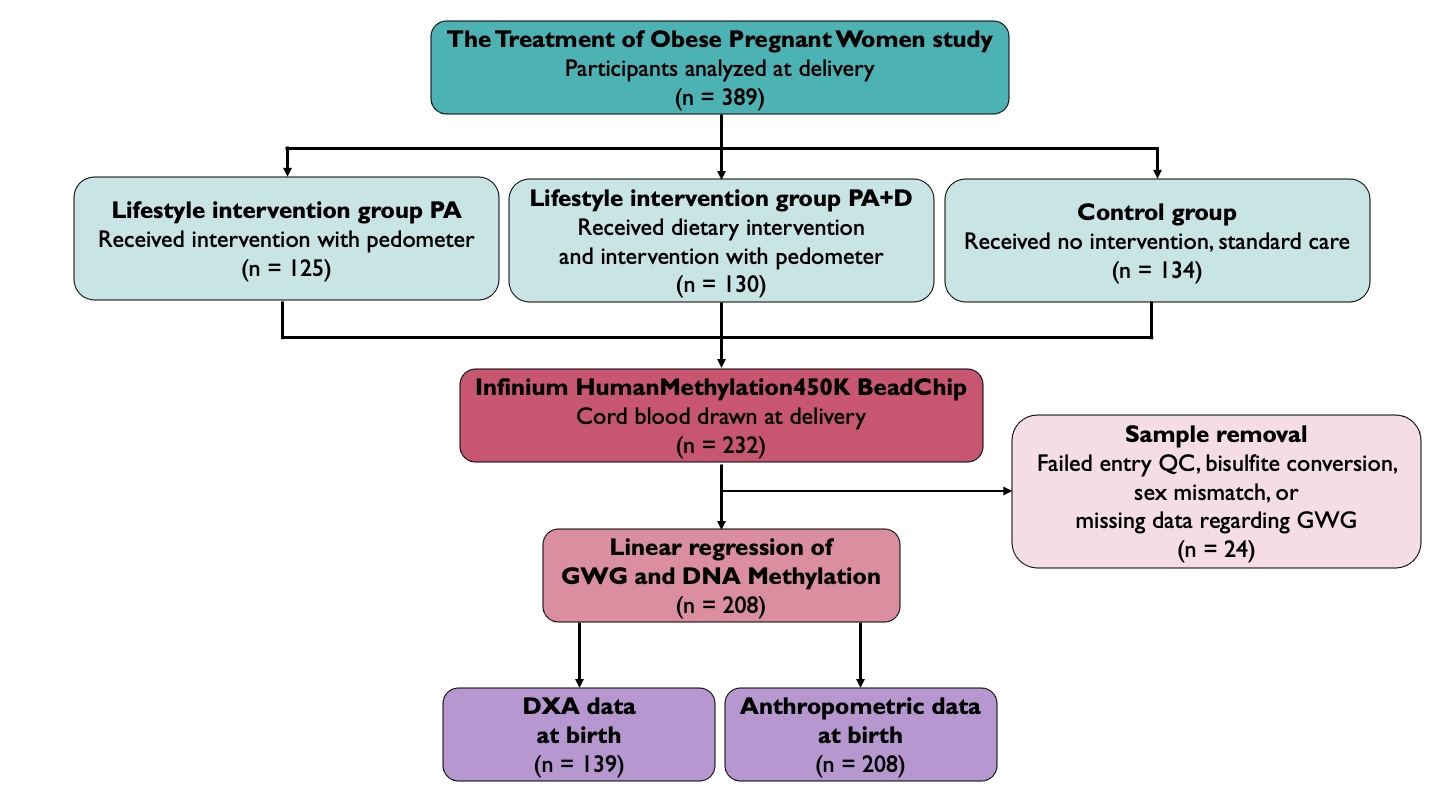

Supplement: Supplementary file 3 — Supporting information [file CTM2-13-e1215-s001.tiff]
